# PrairieDogTown and Kinny: lytic environmental bacteriophages of *Arthrobacter globiformis*

**DOI:** 10.1128/mra.00402-26

**Published:** 2026-07-30

**Authors:** Melanie Baires, Olivia Kelly, Arya Sreenivas, Amber Morton, Theoren Markusson, George Sander, Libertyann Ward-Polonski, Damian Al Khayatt, Abigail Frank, Sophia German-Kavle, Morgan Reed, Shreya Srivastava, Katie Dale, Elizabeth C. Williams, Sabrina Gregoire, Khushi Rathod, Victoria Gilchrist, Carson Bellew, Christian DeLuna, Francesca Makilan, Austen Rowell, Fahareen B. Mosharraf, Adam D. Rudner, Allie C. Smith, Lisa M. Bono

**Affiliations:** 1 TrUE Scholars Program, Center for Transformative Undergraduate Experiences, Texas Tech University 6177, Lubbock, Texas, USA; 2 Program in Translational and Molecular Medicine and Ottawa Institute of Systems Biology and Department of Biochemistry, Microbiology and Immunology, Faculty of Medicine, University of Ottawa 6363, Ottawa, Ontario, Canada; 3 Teaching, Learning, and Professional Development Center, University Library, Texas Tech University 6177, Lubbock, Texas, USA; 4 Department of Biological Sciences, Texas Tech University 6177, Lubbock, Texas, USA; 5 Honors College, Texas Tech University 6177, Lubbock, Texas, USA; Portland State University, Portland, Oregon, USA

**Keywords:** environmental bacteriophage, *Arthrobacter* spp., SEA-PHAGES, phage isolation, genome assembly

## Abstract

Two bacteriophages, PrairieDogTown and Kinny, were isolated from topsoil using *Arthrobacter globiformis* as a host. PrairieDogTown has a 36.2-kb genome encoding 52 genes, whereas Kinny has a 56.4-kb genome with 93 genes. Comparative genomic analysis places PrairieDogTown in Cluster FO and Kinny in Cluster AU in the Actinobacteriophage database.

## ANNOUNCEMENT

We isolated bacteriophage on *Arthrobacter globiformis* B-2979, an adaptable host capable of rapid growth across diverse environments (1). Phages PrairieDogTown and Kinny were isolated from topsoil collected at Texas Tech University in Lubbock, Texas, and the University of Ottawa, Canada, respectively. Soil was collected to the 15 mL mark of a conical tube, mixed with PYCa medium, and incubated with shaking at 30°C and 250 rpm for 1 hour for PrairieDogTown. Soil samples were filled to 5–7.5 mL, submerged beneath 2–3 mL of PYCa medium, and incubated for 2 hours at 220 rpm at 30°C for Kinny. Serial dilutions preceded plaque isolation. Samples were centrifuged at 2,000 × *g* for 10 minutes to pellet the soil for PrairieDogTown; Kinny samples were spun at 3,000 × *g* for 5 minutes. Samples were filtered through a 0.22 µm filter (1).

For Kinny, filtrates were mixed with 3 mL top agar and plated to isolate plaques. Since Kinny was isolated without enrichment, samples were amplified and purified using polyethylene glycol (30% PEG 8000, 3.3M NaCl) precipitation by incubating at 4°C for 4 hours and centrifuging at 15,000 × *g* for 30 minutes. The pellet was resuspended in 15 mM EDTA with 0.5% SDS. PrairieDogTown samples were enriched by incubating with *A. globiformis* for 2–5 days at 30°C and 220 rpm before filtration. Selected plaques were resuspended in phage buffer, triple plaque purified, and used to generate high-titer lysates with >10^8^ PFU/mL (2).

Genomes were extracted from the high-titer lysates by treatment with proteinase K, DNase, and RNase (1). For Kinny, DNA was extracted using phenol/chloroform and ethanol precipitation (1). Concentration and purity were assessed using NanodropOne and QuBit4. For PrairieDogTown, we extracted DNA from the lysate using the QuantaBio Extracta Plus kit and quantified with a BioTek Take3 plate. Both libraries were prepared using NEB Ultra II FS kits (v.3 reagents) (3) and sequenced on an Illumina NextSeq 1000 to generate 100 bp single-end reads (3).

**Fig 1:**
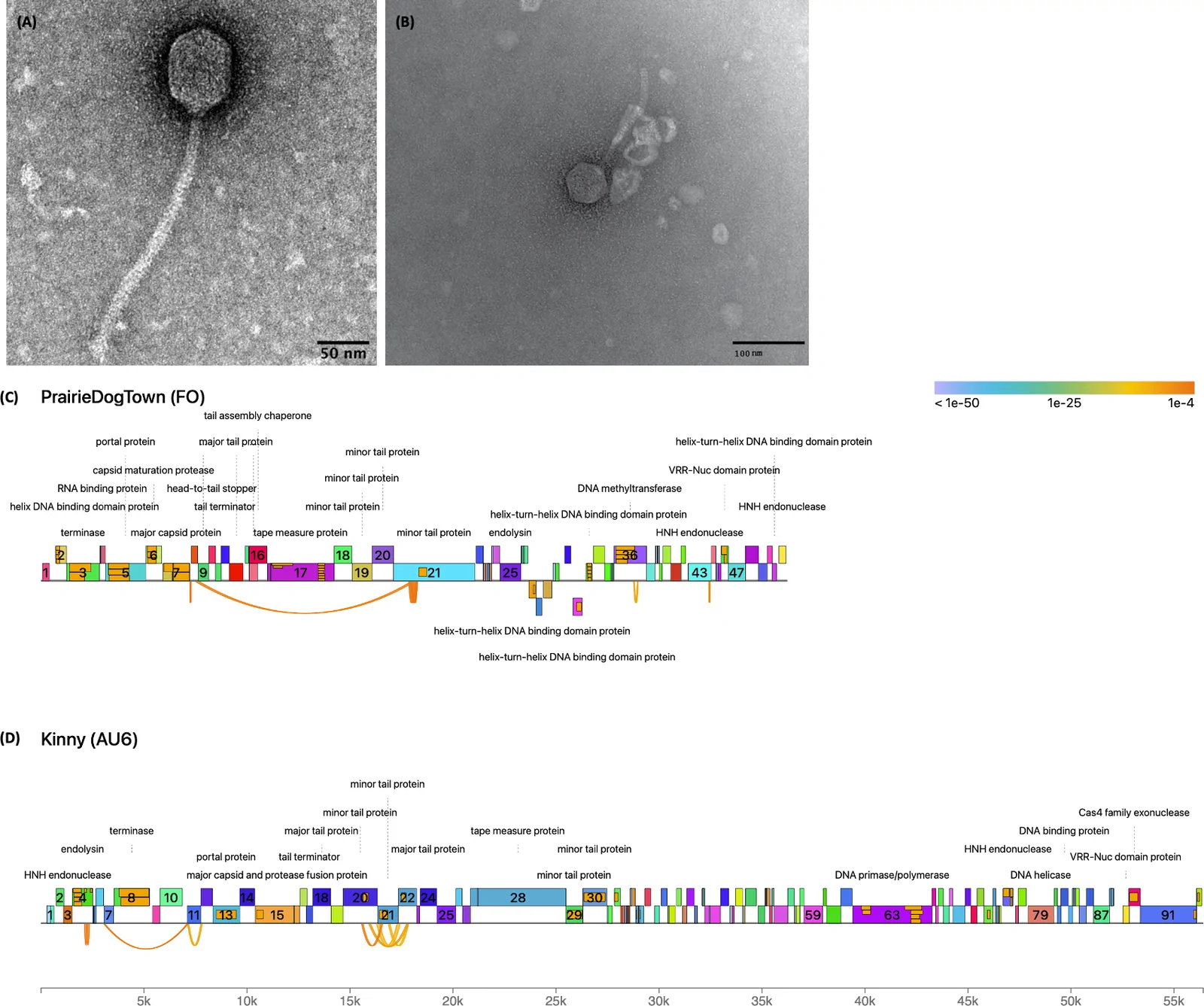
Electron micrograph of the tailed phages (**A**) Kinny and (**B**) PrairieDogTown. Measurements were quantified with ImageJ. Genome maps of (**C**) PrairieDogTown and (**D**) Kinny, with predicted protein-coding genes, shown in the direction of transcription and annotated gene functions labeled. The arcs below the map indicate the pairwise local alignments, with the color indicating the *E* value (see the heatmap in the legend). Genome maps were generated using Phamerator (4).

Lysates were stained with 1% uranyl acetate and visualized using transmission electron microscopy at 100 kV (Hitachi H-7650; Hitachi, Tokyo, Japan) for PrairieDogTown and at 120 kV (JEOL JEM-1400Flash) for Kinny (Fig. 1). Both phages exhibited a Siphoviridae morphotype.

Genomes were assembled using Newbler v2.9 and Consed v29 (3, 5). Gene positions and functions were initially predicted using Glimmer v3.02 (6) and GeneMark v2.5 (7) and manually validated in DNAMaster v5.23.3 (8). Structural annotation was completed using PECAAN (9). A consensus of gene functions was then assigned using BLAST v2.11.01 (Actinobacteriophage and NCBI non-redundant database), Phamerator (4) using Actino_draft database v578), HHpred v3.0 (using PDB_mmCIF70), Pfam v36, and NCBI Conserved Domain Database v3 (4, 10–14). Default settings were used.

PrairieDogTown was assigned to cluster FO using Phamerator (4) based on ≥68% gene content similarity to phages in PhagesDB (11, https://phagesdb.org). All FO members infect *Arthrobacter* and have an average genome length of 37,328 bp with 57.4 genes (11). PrairieDogTown has a GC content of 67.8%, a 36,233 bp genome, and 52 predicted genes (Fig. 1, Table 1). Kinny was assigned to cluster AU using Phamerator (4) based on ≥50% gene content similarity to PhagesDB (11). All AU members infect *Arthrobacter* and have an average genome length of 57,076 bp with 91.6 genes (11). Kinny has a GC content of 52.3%, a 56,422 bp genome, and 93 predicted genes (Fig. 1, Table 1). No tRNAs were detected in the genome of either phage, which was confirmed using tRNAscan-SE v2.0 and ARAGORN. v1.2.38 (15, 16).

**TABLE 1:** Characterization of the reported phages PrairieDogTown and Kinny

Parameter	Data for the isolated phages	
	PrairieDogTown	Kinny
GPS coordinate	33.58793, −101.87309	45.402111, −75.649388
Year found	2024	2024
Genome size (bp)	36,233	56,422
Total number of reads generated	2,371,973	1,821,066
Sequencing coverage	3,431×	2,815×
GC content (%)	68	52
No. of predicted genes	52	93
No. of tRNAs	0	0
No. of hypothetical proteins of unknown function	26	73
Capsid width (nm)	58	60
Capsid length (nm)	52	90
Tail length (nm)	153	251
Facility performing sequencing	Pittsburgh Bacteriophage Institute	Pittsburgh Bacteriophage Institute
GenBank accession no.	PV876925	PV915870
BioProject accession no.	PRJNA488469	PRJNA488469
SRA accession no.	SRP159164	SRP159164

## Data Availability

This project has been deposited in GenBank under accession numbers PV876925 for PrairieDogTown and PV915870 for Kinny.
